# ASSOCIATION BETWEEN PATIENT PORTAL ACTIVITIES AND END-OF-LIFE OUTCOMES

**DOI:** 10.1093/geroni/igad104.0244

**Published:** 2023-12-21

**Authors:** Jennifer Portz, Shaowei Wan, John Powers, Ted Palen

**Affiliations:** University of Colorado, Aurora, Colorado, United States; University of Colorado, Aurora, Colorado, United States; Kaiser Permanente Colorado, Aurora, Colorado, United States; Kaiser Permanente Colorado, Aurora, Colorado, United States

## Abstract

Examining end-of-life (EOL) patient outcomes associated with patient portal use will help identify benefits and barriers of portal use during EOL care. The primary objective of this retrospective cohort study was to assess the association between EOL outcomes and patient portal use among a cohort (N=6,517) of deceased patients (18+ years old) in the last 12 months of life within Kaiser Permanente Colorado (KPCO). Outcomes including 1) number of hospitalizations in the last 30 days of life, 2) having documented advance directive before death, and 3) dying in hospice were investigated by levels of patient portal use (no use, non-active use, active use without provider, and active use with a provider) for the first six months of the last year of life. A generalized linear model with Poisson distribution, a log link and an offset to account for person-days of follow-up was used to estimate rates of hospitalizations in the last 30 days. Logistic regression was used to estimate advance directive and death in hospice outcomes. Patient portal use appeared to be associated with beneficial EOL outcomes such as advance care planning (P = 0.0226) and dying in hospice (P = 0.0070). Hospitalizations were greater with higher levels of patient portal use (P = 0.0492), suggesting that patients/caregivers may have used patient portals for disease related information and accessing health services during times of need. More research is needed to leverage patient portal use to intentionally engage, educate and guide patients with serious illness and approaching EOL.

